# Identification of Critical Signature in Post‐Traumatic Stress Disorder Using Bioinformatics Analysis and in Vitro Analyses

**DOI:** 10.1002/brb3.70243

**Published:** 2025-01-19

**Authors:** Lifen Liu, Yang Liu, Rui Li, Yue Teng, Shuyang Zhao, Jinhong Chen, Changjiang Li, Xinyu Hu, Lin Sun

**Affiliations:** ^1^ School of Psychology Shandong Second Medical University Weifang Shandong People's Republic of China; ^2^ Department of Bioscience and Technology Shandong Second Medical University Weifang Shandong People's Republic of China; ^3^ College of Extended Education Shandong Second Medical University Weifang Shandong People's Republic of China; ^4^ CAS Key Laboratory of Mental Health, Institute of Psychology Chinese Academy of Sciences Beijing People's Republic of China; ^5^ Department of Psychology University of Chinese Academy of Sciences Beijing People's Republic of China; ^6^ Management Committee of Shanting Economic Development Zone Zaozhuang Shandong People's Republic of China; ^7^ Department of Neurosurgery Shanting District People's Hospital Zaozhuang Shandong People's Republic of China; ^8^ Faculty of Psychology Southwest University, Chongqing China; ^9^ Department of Psychological Counseling Center Weifang Mental Health Center, Weian Road, Weifang, Shandong China

**Keywords:** bioinformatics, DEGs, microarray, PTSD

## Abstract

**Background:**

Post‐traumatic stress disorder (PTSD) is a complex psychiatric condition that emerges following exposure to trauma and significantly affects daily functioning. Current research is focused on identifying effective treatments for PTSD. Advances in bioinformatics provide opportunities to elucidate the underlying mechanisms of PTSD.

**Methods:**

RNA sequencing (RNA‐seq) datasets were retrieved from the Gene Expression Omnibus (GEO) database. Differentially expressed genes (DEGs) were identified using GEO2R. Weighted gene co‐expression network analysis (WGCNA) was employed to examine gene correlation patterns. Gene ontology (GO) and Kyoto Encyclopedia of Genes and Genomes (KEGG) pathway analyses were performed for functional annotation and enrichment analysis, respectively. The MCODE plugin in Cytoscape software was utilized to analyze the protein–protein interaction (PPI) network. Anxiety and depression in a mice stress model were assessed using the open‐field test (OFT), elevated plus maze test (EPMT), and forced swimming test (FST). Real‐time quantitative PCR (qRT‐PCR) was conducted to validate key genes in stress‐exposed models.

**Results:**

A total of 157 common upregulated DEGs and 53 common downregulated DEGs were identified in the amygdala (AMY) and the hippocampus (HIP). Notably enriched pathways included neuroactive ligand‐receptor interaction, mechanistic target of rapamycin (mTOR) signaling pathway, nicotine addiction, and dopaminergic synapse. The PPI network identified four hub genes, with key pathways associated with nicotine addiction and dopaminergic synapse. qRT‐PCR validation confirmed that the expression trends of these four genes were consistent with microarray data. Behavioral tests (OFT, EMPT, and FST) revealed significant changes.

**Conclusion:**

This study utilized bioinformatics and in vitro experiments to identify genes and pathways potentially crucial for PTSD development. Key genes were validated in a mouse model, providing insights into potential target genes for PTSD treatment.

## Abstract 2

Through bioinformatics analysis, we show that multiple genes and related pathways may be key mechanisms in the development of PTSD, one of the few methods currently used in PTSD research, laying the foundation for obtaining potential therapeutic targets related to PTSD.

## 1 Introduction

Post‐traumatic stress disorder (PTSD) is a severe psychiatric disorder that can develop months or even years after exposure to intense trauma (A.P. Organization 2013). The core symptoms of PTSD include recurrent trauma‐related experiences, avoidance of trauma cues, and hyperarousal. According to the World Health Organization, over 70% of individuals experience at least one traumatic event in their lifetime, with a lifetime prevalence of PTSD estimated at 3.9% (Benjet et al. 2016). PTSD is challenging to treat, persists long‐term, and not only affects mental well‐being but also contributes to various physiological disorders, significantly impairing the quality of life of patients. To date, PTSD‐pertinent mechanism studies include heredity, neuroendocrine, immune system, and neurocircuits; however, the precise pathological mechanisms remain unclear. In addition, structural and functional abnormalities in the brain may also influence PTSD development, making it a current focus of research into PTSD pathogenesis.

Recent studies have identified that brain circuits involving the amygdala (AMY), the hippocampus (HIP), and the medial prefrontal cortex (mPFC) are implicated in stress responses. The AMY plays a crucial role in the learning and expression of fear, threat detection, and emotional memory (Tanaka et al. 2019). Structural and functional abnormalities in the AMY are significantly associated with stress‐induced PTSD (Ahmed‐Leitao et al. 2016; Ding, Han, and Shi 2010; Liu et al. 2014). The HIP, a key component of the brain's limbic system, is linked to anxiety and negative emotions. Research indicates that chronic stress in PTSD leads to injury of the HIP (Guo et al. 2012). Moreover, the formation of fear memory is strongly associated with changes in synaptic plasticity, with the HIP and AMY working together to establish this memory. The synaptic plasticity of the HIP is crucial for retrieving memory signals and transmitting them to the AMY (Honey and Good 1993). In addition, stress has been shown to induce gene‐level alterations in both the HIP and AMY (Lori et al. 2019). This study focuses on examining the AMY and HIP through bioinformatic analysis.

In recent years, biomedical data analysis has become central to bioinformatics, facilitating research into the molecular mechanisms of diseases and the development of individualized treatments for challenging conditions (Lebo et al. 2020). Microarray analysis and next‐generation sequencing (NGS) have emerged as crucial tools in non‐oncology medicine. Advances in sequencing technology have led to the identification of genes and pathways associated with PTSD. Research has predominately focused on RNA‐seq of peripheral blood from patients with PTSD, revealing downregulation of FK506 binding protein 5 (*FKBP5*) and signal transducer and activator of transcription 5B (*STAT5B*), both of which are involved in regulating inflammatory and glucocorticoid responses (Binder 2009; Maddox, Schafe, and Ressler 2013). However, while blood samples are more accessible for gene expression studies, they have limitations for brain disorders. Postmortem brain transcriptomics in patients with PTSD offers a more direct approach to uncovering the complex molecular mechanisms underlying PTSD. PTSD postmortem studies have identified upregulation of the multifunction protein p11 and highly regulated genes like serum/glucocorticoid‐regulated kinase 1 (*SGK1*) (L. Zhang et al. 2008; Licznerski et al. 2015). Nonetheless, there are few such studies due to the lack of organized repositories for postmortem PTSD brain tissue. Therefore, to address this limitation, brain transcriptomics in animal models can provide valuable insights. For instance, an animal study showed that the transcripts FKBP5 and brain‐derived neurotrophic factor (BDNF) of the social‐stress (SS) mouse model were suppressed in the different brain regions (Muhie et al. 2015). Recent research has primarily focused on the hypothalamus–pituitary–adrenal (HPA) axis, but exploring other mechanisms is crucial. To this end, we analyzed mRNA expression microarray datasets from the Gene Expression Omnibus (GEO), a repository established by the National Center for Biotechnology Information (NCBI) that provides high‐throughput functional genomic data (Barrett et al. 2013).

This study employs bioinformatics analysis to explore potential mechanisms and biomarkers of PTSD. Using the SS mouse model, we selected a dataset and validated key genes through real‐time quantitative PCR (qRT‐PCR) experiments to determine their regulation in stress‐exposed mice exhibiting PTSD‐like symptoms.

## 2 Materials and Methods

### 2.1 Materials

#### 2.1.1 Experimental Animals

Twelve 6‐week‐old male C57BL/6J and 10‐week‐old male CD1 mice were obtained from the Experimental Animal Center of Weifang Medical University. The C57BL/6J mice were housed individually at 22 ± 2°C and 50% ± 15% humidity under a 12 h light/dark cycle before experimentation. C57BL/6J mice were randomly divided into two groups: the control group (CON) and the social frustration stress group (SS). All experimental procedures adhered to the National Institutes of Health Guidelines (Use of Laboratory Animals) and were approved by the Animal Care and Use Committee of Weifang Medical University.

#### 2.1.2 Screening Aggressive Mice

Prior to the main experiments, CD1 mice were screened for aggression. C57BL/6J mice were introduced into CD1 mouse cages for screening, conducted once every 3 days. An effective attack was defined as an aggression lasting at least 5 s occurring within 1 min. CD1 mice that exhibited at least five effective attacks within two days were classified as aggressive. Nonaggressive CD1 mice were removed from the study, and only aggressive CD1 mice were retained.

#### 2.1.3 Data Accessibility and DEG Identification

mRNA expression data were downloaded from the GEO (https://www.ncbi.nlm.nih.gov/) using PTSD as the search term. The GSE45035 dataset, obtained from the GPL7202 platform (Agilent‐014868 Whole Mouse Genome Microarray 4×44K G4122F) (Muhie et al. 2015), was used for analysis. This dataset includes 230 samples representing transcriptome profiles of the brain and blood from stress‐exposed and control C57BL/6 mice. We analyzed microarray data from the AMY and HIP collected 24 h after 10 days of SS. DEGs were identified using GEO2R, an analysis tool of GEO, with probes lacking gene symbols or containing duplicate symbols excluded. Genes were considered statistically significant if |LogFC (fold change)| > 1 and *p* < 0.05. Data visualization was performed using a volcano plot in R software. Venn2.1 software was used to identify overlapping upregulated and downregulated DEGs between the AMY and the HIP. The interesting genes were selected for further analysis.

### 2.2 Methods of Bioinformatic Analysis

#### 2.2.1 Weighted Gene Co‐Expression Network Analysis

Weighted gene co‐expression network analysis (WGCNA) is used to identify biologically significant co‐expressed gene modules and to explore the relationship between gene networks and disease (Langfelder and Horvath 2008). For this study, we analyzed datasets from GSE45035 to identify modules related to the CON and SS groups. WGCNA was performed using the “WGCNA” package in R software version 4.4.1. Prior to analysis, a hierarchical cluster analysis was performed using the Hclust function in the R software to exclude outlier samples. The “pickSoftThreshold” function in the WGCNA package was used to select a suitable soft threshold *β* (ranging from 1 to 20) to meet the criteria for scale‐free networks. Cluster analysis was then used to identify co‐expression modules, with a minimum module size of 60 genes. The expression profile of each module was obtained by calculating module eigengenes and their correlation with the grouped features.

#### 2.2.2 Kyoto Encyclopedia of Genes and Genomes Enrichment Analysis of DEGs

Overlapping upregulated and downregulated DEGs were analyzed using the R package clusterProfiler. Gene ontology (GO) enrichment analysis was performed to interpret the functional information related to proteins, including molecular functions (MF), biological processes (BP), and cellular components (CC) (Manfredi et al. 2019). The Kyoto Encyclopedia of Genes and Genomes (KEGG) pathway database was used to identify metabolic and signal transduction pathways associated with the genes. KEGG orthology (KO) functional annotation was performed using WEGO (Ye et al. 2006). The enriched metabolic pathways, or signal transduction pathways, of genes were identified based on the KEGG database (Kanehisa et al. 2008).

#### 2.2.3 PPI Network Construction and Module Analysis

The Search Tool for the Retrieval of Interacting Genes/Proteins (STRING) was used to predict the protein–protein interaction (PPI) network for DEGs (Manfredi et al. 2019). Data visualization and analysis were conducted using Cytoscape software. Subsequently, close‐knit modules within the PPI network were identified using the Molecular Complex Detection (MCODE) plugin.

### 2.3 Methods of animal model analysis

#### 2.3.1 Modeling Social Frustration Stress

Social frustration stress was modeled following a previously described method (Golden et al. 2015). The screened CD1 attack mice were placed in a cage with a transparent partition containing holes. The CON group C57BL mice were housed on another side of the partition and fed for 24 h. The SS group C57BL were then placed on the same side of the cage as CD1 mice and subjected to 5–10 min of aggression, ensuring no injury occurred. After the aggression, SS C57BL mice were returned to their original position and allowed to see and smell the CD1 mice for 24 h. This procedure was repeated daily for ten consecutive days. Following the final social stress experiment, C57BL mice were housed individually in standard mouse cages, and behavioral tests were performed 24 h later.

#### 2.3.2 The Open‐Field Test

After 10 days of stress exposure, directly or indirectly, the attacked mice were rested for one day. Open‐field test (OFT) was used to evaluate anxiety behavior. The subject mice were placed in a wide, enclosed box (50 cm × 50 cm × 50 cm) and allowed to explore freely. Behavior was recorded and analyzed using the Smart‐3.0 video tracking system. Mice were placed in the center of the arena and observed for 5 min. The distance traveled, time spent, and number of entries into the center were measured.

#### 2.3.3 Elevated Plus Maze Test

Elevated plus maze test (EPMT) is a widely used model for evaluating anxiety‐like behavior. The maze consists of an elevated plus‐shaped platform with two closed arms (30 cm × 30 cm) and two open arms (30 cm × 30 cm). Each mouse was placed in the central square of the maze facing one of the open arms. The test duration was 5 min, and behavior was recorded using the Smart‐3.0 video tracking system. After each trial, the appliance was disinfected with 75% ethanol.

#### 2.3.4 Forced Swimming Test

Forced swimming test (FST) assessed despair‐like behavior. Mice were placed in transparent acrylic drums (diameter = 20 cm, water depth = 25 cm) filled with water at 25 ± 1°C for 5 min and 30 s. Immobility time, defined as floating with only the head above the water, was recorded. Immobility time was quantified by a researcher blinded to the experimental groups.

#### 2.3.5 Real‐Time Quantitative PCR

Mice were euthanized using narcotics, and their brains were rapidly extracted. The AMY and HIP were isolated. Then, 100 µL of TRIzol reagent was added to the extracted brain tissue, and the tissue was crushed until clear and transparent. Following this, 200 µL of chloroform was added to the sample, centrifuged, and the upper aqueous phase was aspirated. An equal volume of isopropanol was added to remove the proteins, and, after centrifugation, a white precipitate was visible at the bottom or side walls of the tube. The supernatant was discarded, and the precipitate was washed with 1 mL of 75% ethanol, followed by centrifugation and supernatant discarding. The 75% ethanol washing was repeated, and the RNA precipitate was dried naturally. Afterward, 30 µL of enzyme‐free water was added to dissolve the RNA precipitate, and the resulting samples were stored at −80°C. RNA quantity and quality were measured using a nucleic acid protein detector. cDNA was synthesized using the PrimeScript RT Reagent Kit with gDNA Eraser (RR047A TaKaRa, China). According to the manufacturer's instructions, qRT‐PCR was accomplished using the SYBR Premix Ex TaqTM Kit (TaKaRa) on a QuantStudio7 Flex biosystem (Thermo Fisher Scientific, Waltham, MA, USA) with 40 cycles. GAPDH was used as an internal reference control. The comparative *C*_T_ method (2−ΔΔ*C*_T_ method) was used to determine the relative mRNA abundance (Livak and Schmittgen 2013). All statistical data are presented as mean ± standard error (the standard error of mean [SEM]). Differences between groups were analyzed using an independent *t*‐test. Primer sequences used for amplification are listed in Table 1.

**TABLE 1:** qRT‐PCR primer sequence list.

Gene symbol	Forward (3ʹ–5ʹ)	Reverse (3ʹ–5ʹ)
GAPDH	CTACGAGGGCTATGCTCTCC	TTTGATGTCACGCACGATTT
GABRA1	ACTGCTGGACGGTTATGACAATCG	GGTCTGAAACTGGTCCGAAACTGG
GABRA2	AGAGAATCGGTGCCAGCAAGAAC	AAGCCACTTTCGGGAGGGAATTTC
GRIN2B	GCAAGCCTGGCATGGTCTTCTC	GGAGCAAGCGTAGGATATTGGAGTG
DRD2	TGTATCATTGCCAACCCTGCCTTC	GACGCTTGCGGAGAACGATGTAG

#### 2.3.6 Statistical Analyses

The data of OFT, FST, and qRT‐PCR data were demonstrated by SPSS19.0 statistical analysis software and were analyzed by applying the *t*‐test. Significant differences are defined as follows: *p < 0.05; **p < 0.01; ***p < 0.001.

## 3 Results

### 3.1 Construction of WGCNA

**FIGURE 1:**
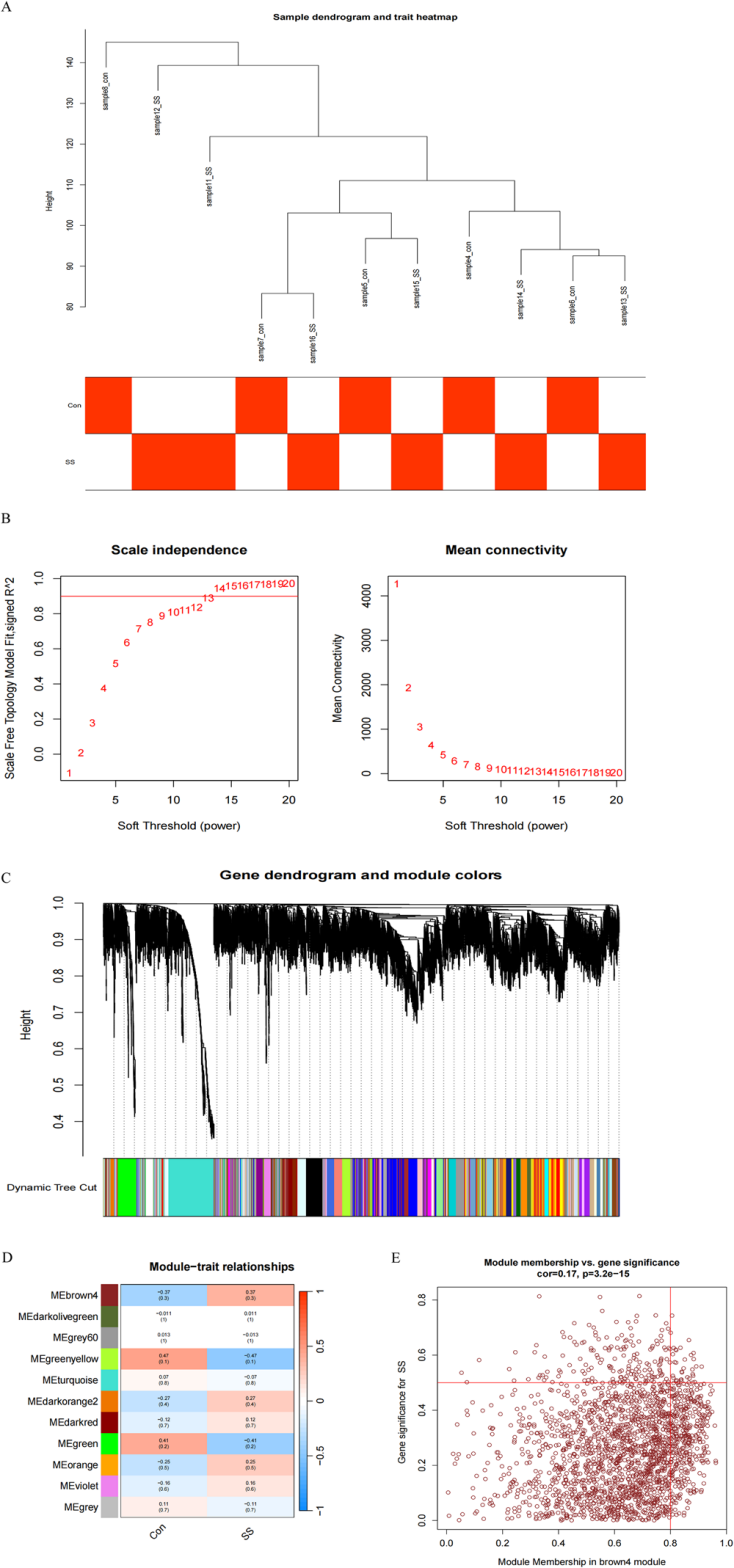
Identification of gene modules associated with the SS model by WGCNA. (A) Sample clustering for WGCNA and detection of anomalous samples. (B) Selection of a suitable soft threshold. The left‐hand panel shows the variation of the fitting exponent for different soft‐threshold powers, and the right‐hand panel shows the average connections corresponding to different soft thresholds. (C) Hierarchical clustering tree diagram of identified co‐expressed genes, with different colors representing the corresponding modules. (D) Heat map of the relationship between each gene module and the PTSD model, with red representing positive correlation and blue representing negative correlation. (E) Scatter plots of GS and MM in corresponding modules.

Outliers in the dataset were removed based on the clustering tree of samples (Figure 1A). The optimal soft threshold was selected to ensure that the gene distribution adhered to a scale‐free network (Figure 1B). A dendrogram of gene modules was generated by WGCNA, identifying 11 modules (Figure 1C). Correlation analysis between modules and the PTSD model revealed that the brown module had the highest correlation score in the module–modeling relationship plot (Figure 1D). Figure 1E illustrates the significant correlation between gene significance (GS) and module membership (MM) for the brown modules in the selected gene set.

### 3.2 Expression of DEGs

**FIGURE 2:**
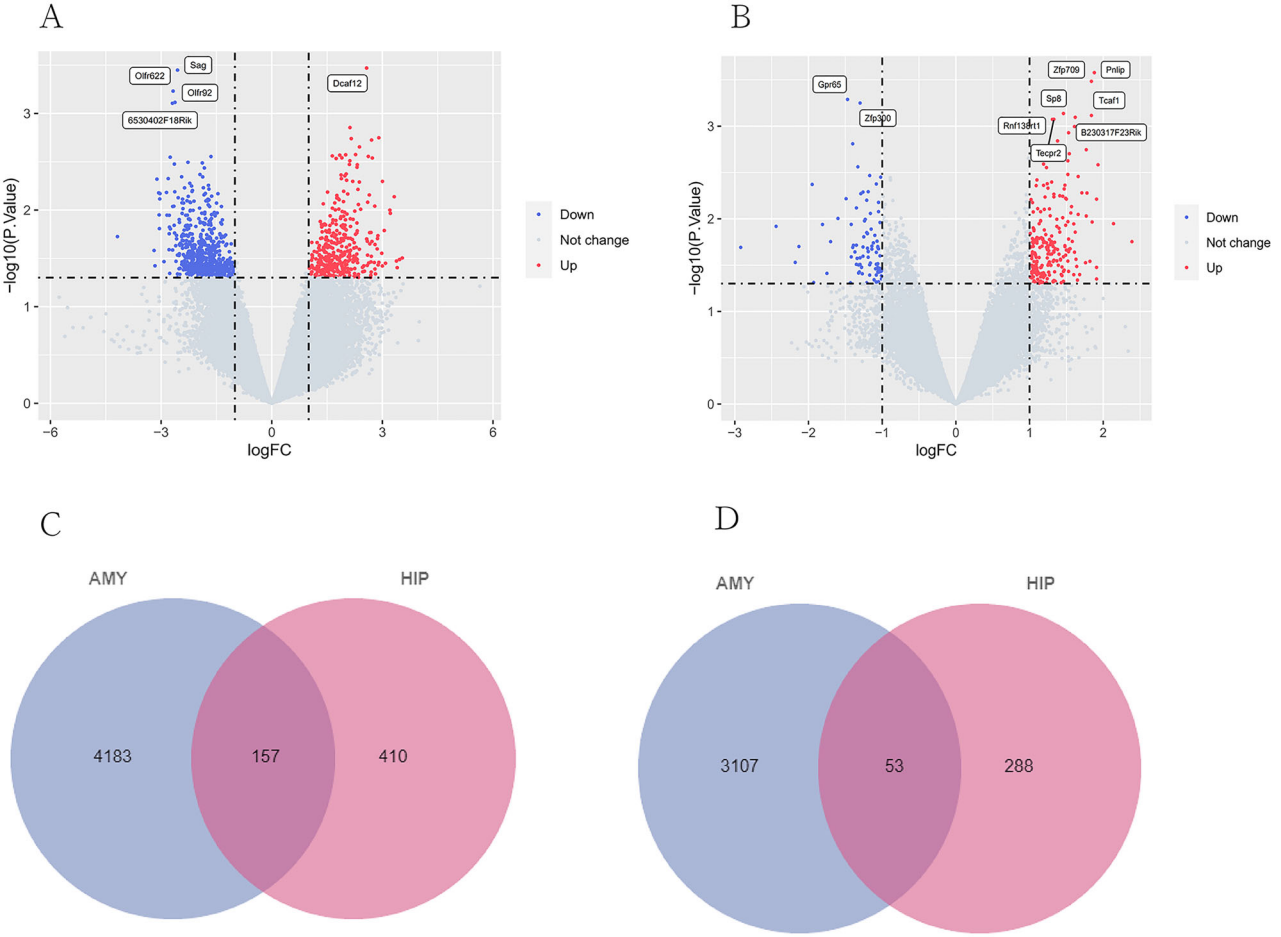
Identification of DEGs shared in the AMY and HIP. Venn diagrams of common DEGs between HIP and AMY. (A) The volcano map of GSE45035 in AMY. Blue plots as downregulated genes, red plots as upregulated genes, and grey plots as genes with no significant difference. (B) The volcano map of GSE45035 in the HIP. (C) Overlapping upregulated differentially expressed genes. (D) Overlapping downregulated differentially expressed genes.

Differential expression analysis revealed 4183 upregulated DEGs and 3107 downregulated DEGs in the AMY and 410 upregulated DEGs and 288 downregulated DEGs in the HIP, as shown in Figure 2A,B. The intersection of DEGs between AMY and HIP, identified using the Venn online tool, included 157 common upregulated DEGs and 53 common downregulated DEGs (Figure 2C,D).

### 3.3 Functional Annotations

**FIGURE 3:**
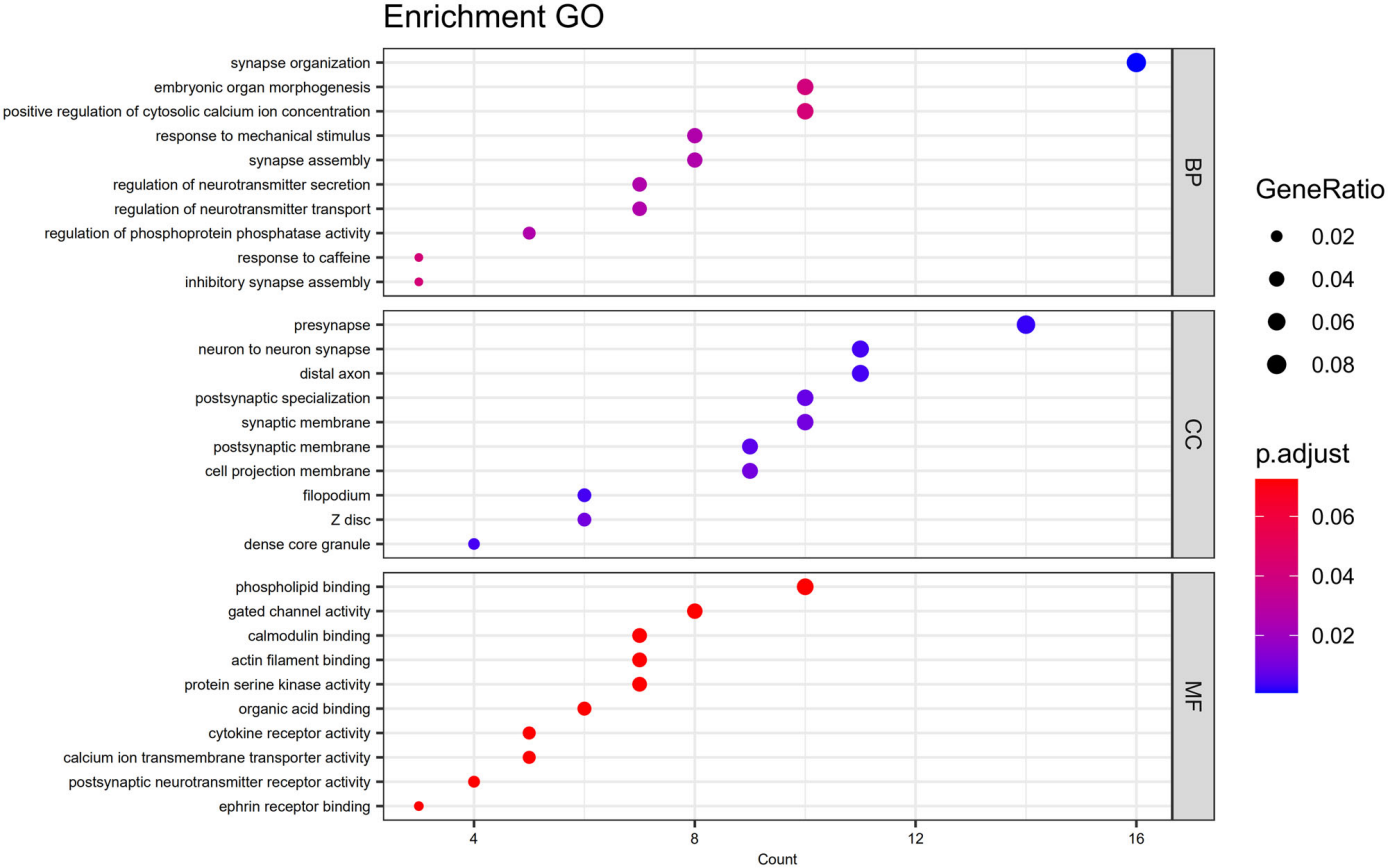
GO analysis of DEGs. BP, biological process; CC, cellular component; MF, molecular function. The horizontal coordinate is count, the number of genes of interest annotated in the entry. The vertical coordinate represents each GO comment entry. Gene ratio, which is the ratio of the number of genes in the entry to all genes. The color of the dots represents the *q* value of the hypergeometric test.

**FIGURE 4:**
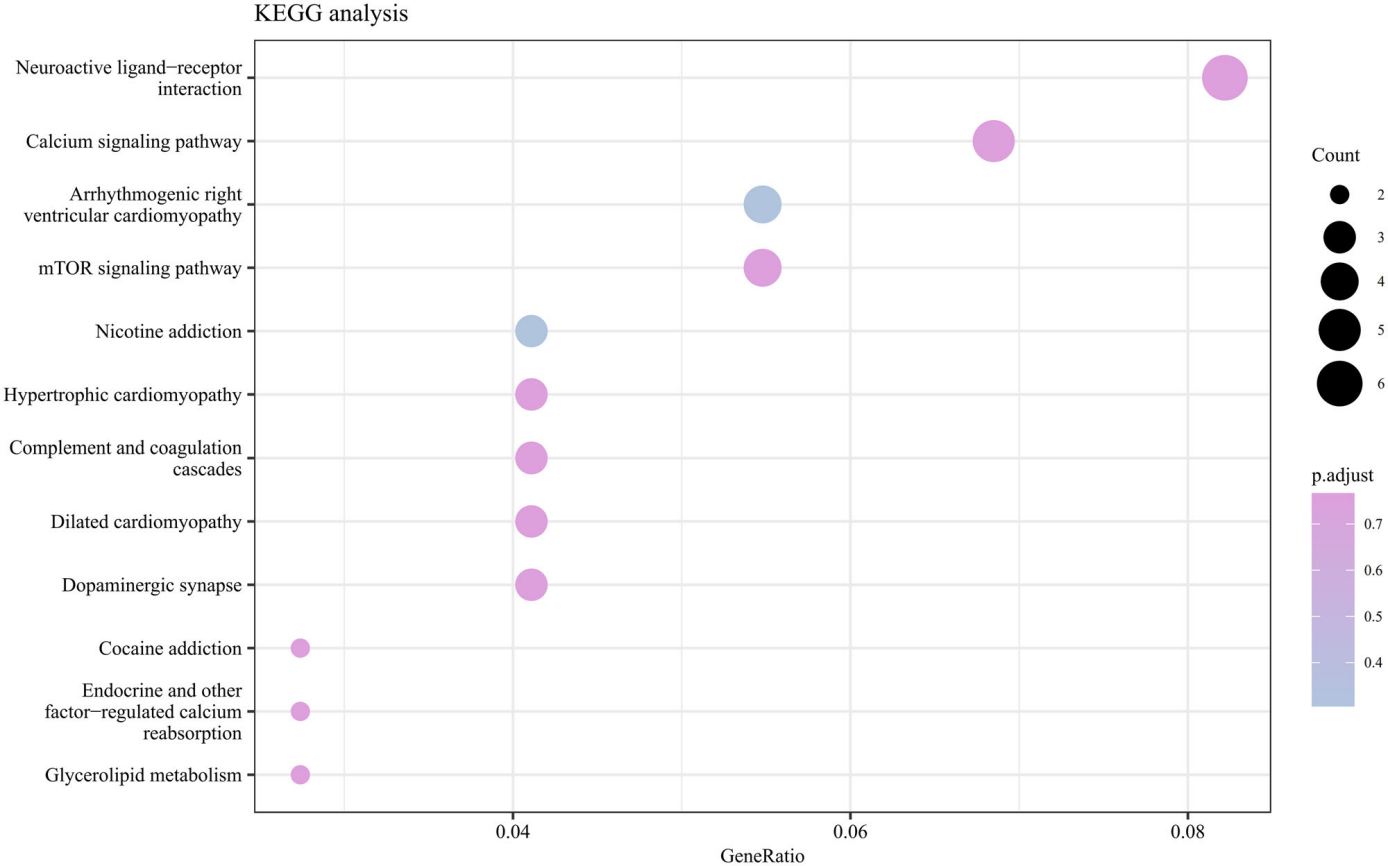
KEGG pathway of DEGs. The horizontal coordinate is GeneRatio. The ratio of genes of interest annotated in the entry to the number of all differentially expressed genes, and the vertical coordinate is each pathway entry. The size of the dot represents the number of the dot size represents the number of differentially expressed genes annotated in the pathway, and the dot color represents the p.adjust of the hypergeometric test.

GO analysis of the DEGs indicated insignificant enrichment in BPs related to synapse organization, embryonic organ morphogenesis, and positive regulation of calcium ion concentration. CC enrichment included presynapse, neuron‐to‐neuron synapse, and distal axon. MF enrichment was primarily associated with phospholipid binding and gated channel activity. Furthermore, these results were visualized by R (Figure 3). KEGG pathway analysis revealed that DEGs were significantly enriched in the neuroactive ligand‐receptor interaction, mTOR signaling pathway, nicotine addiction, and dopaminergic synapse (Figure 4).

### 3.4 Screening of Hub Genes

**FIGURE 5:**
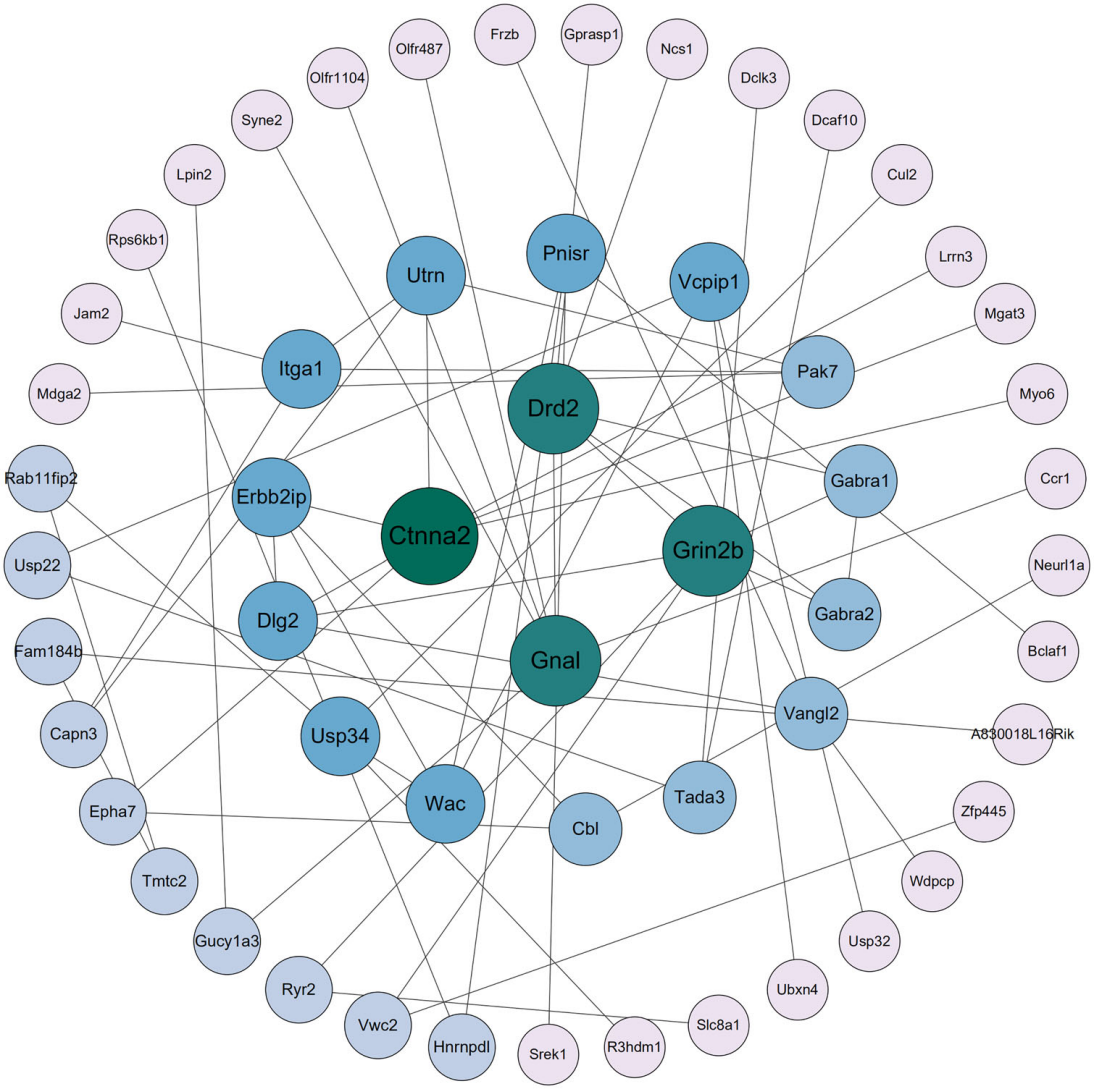
The differential expressed protein–protein interaction network. Proteins are represented by circles, and interactions were presented with edges. The size of the circle represents betweenness centrality.

For PPI relationships among overlapping DEGs were analyzed using STRING. A total of 55 nodes and 61 edges were analyzed with Cytoscape (Figure 5). Two clusters were identified using the MCODE plugin. Four hub genes, based on the degree of connectivity, were identified: gamma‐aminobutyric acid type A receptor subunit alpha 1 (*Gabra1*), gamma‐aminobutyric acid type A receptor subunit alpha 2 (*Gabra2*), dopamine receptor D2 (*Drd2*), and glutamate receptor, ionotropic, NMDA2B (epsilon 2) (*Grin2b*).

### 3.5 SS‐Induced Anxiety‐Like Behaviors

**FIGURE 6:**
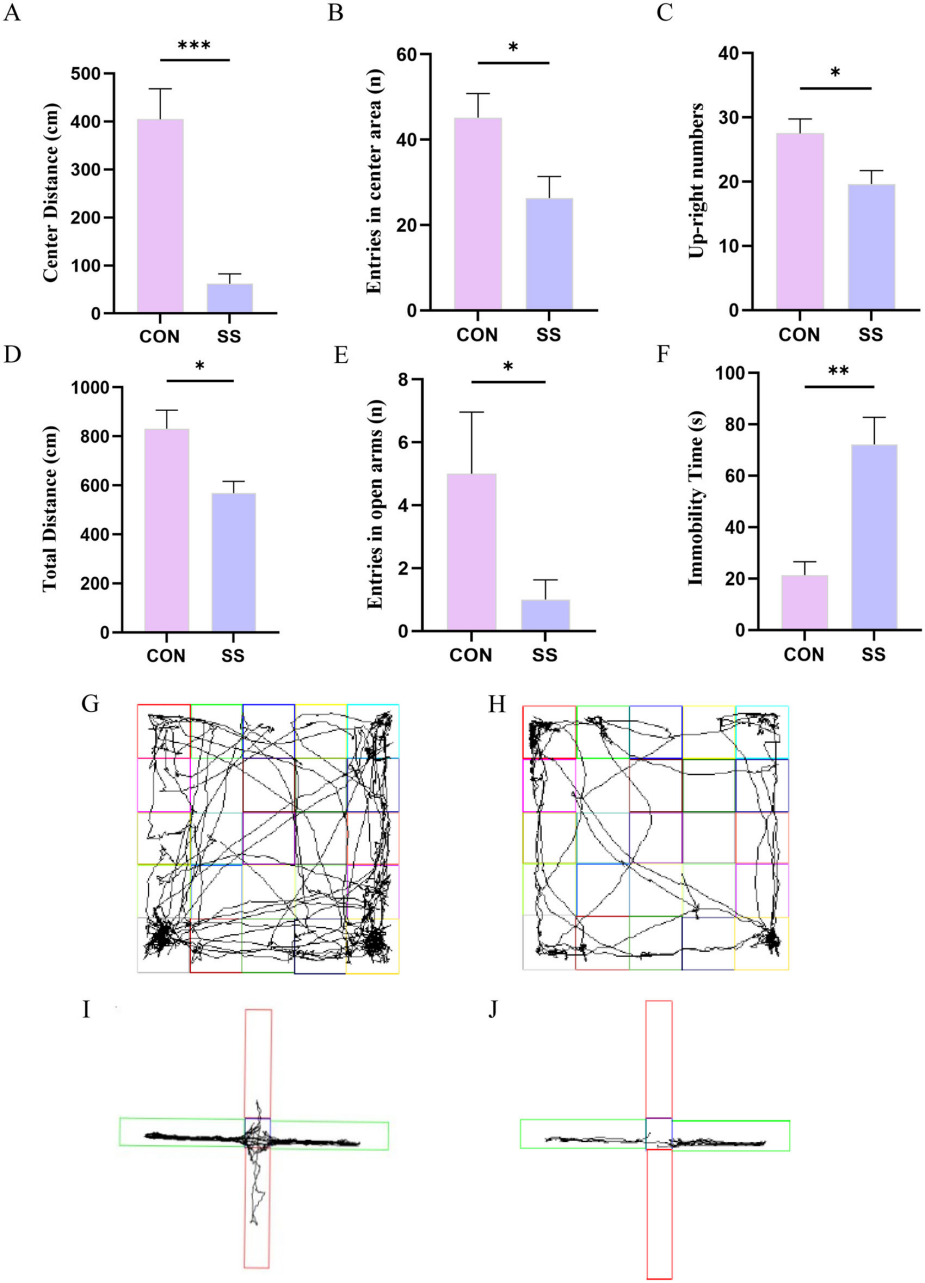
Effects of the SS procedure on the behavior of mice (*n* = 6). (A) The center distance in the OFT of mice in each group (*t* = 5.129; *p* = 0.000). (B) The entries of the center area in the OFT of mice in each group (*t* = 2.490; *p* = 0.032). (C) The number of upright in the OFT of mice in each group (*t* = 2.560; *p* = 0.023). (D) The total distance in all areas in the EPMT of mice in each group (*t* = 2.946; *p* = 0.015). (E) The entries in the open arms in the EPMT of mice in each group (*z* = −2.278; *p* = 0.022). (F) Immobilization time for each group (*t* = 4.323; *p* = 0.002). (G) Representative video tracking images of OFT in the CON groups. (H) Representative video tracking images of OFT in the SS groups. (I) Representative video tracking images of EPMT in CON groups. (J) Representative video tracking images of EPMT in SS groups. Data are represented as mean ± SEM, *p < 0.05, **p < 0.01, and ***p < 0.001.

As shown in Figure 6, SS group mice exhibited reduced center area movement distance, fewer entries into the center area, and decreased upright posture compared to the CON group in the OFT. In the EMPT, SS group mice displayed significant anxiety‐like behaviors, evidenced by fewer entries into the open arms and reduced total distance traveled. A significant increase in immobilization time in the FST was also observed in the SS group.

### 3.6 Validation of Hub Genes by qRT‐PCR

**FIGURE 7:**
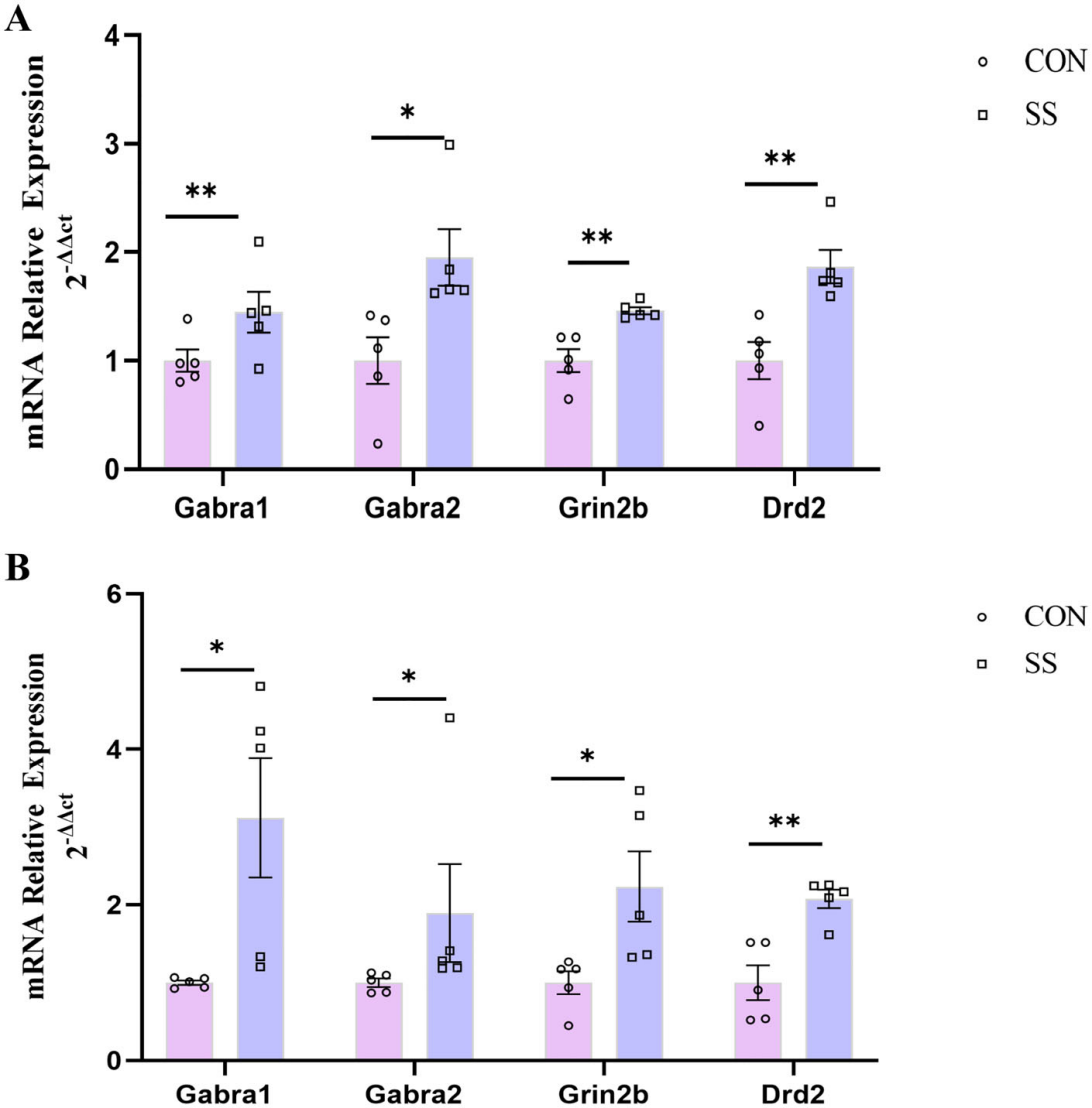
The results of the qRT‐PCR (*n* = 5). (A) The mRNA relative expression level of HIP: Gabra1 (*t* = 3.407; *p* = 0.009), Gabra2 (*t* = 2.801; *p* = 0.023), Grin2b (*t* = 4.135; *p* = 0.003), Drd2 (*t* = 3.765; *p* = 0.006). (B) The mRNA expression level of the AMY: Gabra1 (*t* = 2.761; *p* = 0.025), Gabra2 (*t* = 3.110; *p* = 0.014), Grin2b (*t* = 2.597; *p* = 0.032), Drd2 (*t* = 4.273; *p* = 0.003). Data are represented as mean ± SEM, *p < 0.05, **p < 0.01, and ***p < 0.001.

qRT‐PCR was used to validate the relative mRNA expression levels of the hub genes in hippocampal and AMY tissues from both groups (Figure 7). qRT‐PCR results demonstrated that the hub genes *Gabra1*, *Gabra2*, *Grin2b*, and *Drd2* were significantly upregulated in AMY tissue, consistent with the microarray data.

## 4 Discussion

PTSD is a severe psychiatric disorder that can develop after exposure to life‐threatening stressors (Stein and Simon 2021). Due to its complexity, many patients have only a superficial understanding of their condition, and current clinical practice often lacks personalized care based on individual neurobiological profiles. Thus, effective therapies for PTSD remain a critical area of research. A growing body of evidence suggests that dysregulation of nerve signaling molecules in the brain is an important mechanism in the development of PTSD, causing symptoms such as depression‐like, anxiety‐like, and fear‐like symptoms. Therefore, the identification of genes associated with PTSD is important for studying its pathogenesis and providing possible diagnostic markers and therapeutic approaches (Chevalier et al. 2021; Chakraborty et al. 2015). In this study, we aimed to identify potential biological markers and elucidate molecular mechanisms associated with PTSD.

Based on our selected dataset, only one module was filtered to be significantly correlated between GS and MM by WGCNA analysis. According to previous studies on WGCNA analysis, this may be because the dataset we selected is more focused in its orientation and the results obtained are more accurate (Malki et al. 2013). By further analyzing DEGs in HIP and AMY, we screened their up‐regulated and down‐regulated genes and identified the crossover genes among them. In our present research, we found a total of 157 common upregulated DEGs and 53 common downregulated DEGs in the AMY and the HIP. To further explore the key genes in PTSD, we analyzed these genes for GO functional annotation (including BP, CC, and MF) and KEGG pathway enrichment and constructed a protein–protein network.

KEGG pathway analysis revealed that these dysregulated genes were primarily enriched in the neuroactive ligand‐receptor, the mTOR signaling pathway, nicotine addiction, and dopaminergic synapse. Although research on neuroactive ligand‐receptor interactions in PTSD is limited, the mTOR signaling pathway has been implicated in various psychiatric disorders, including PTSD and depression (Ni et al. 2020; Abelaira et al. 2014). Previous studies have shown that the upregulation of the BDNF–mTOR signaling pathway can alleviate depression‐like symptoms and improve spatial learning in the PFC and HIP of PTSD animal models (Ni et al. 2020). Conversely, dichlorination with the inhibitor rapamycin eliminated the antidepressant effect in a PTSD rat model (Li et al. 2010). In addition, acupuncture has been shown to improve PTSD symptoms induced by single‐prolonged stress (SPS) through modulation of the mTOR signaling pathway (Oh et al. 2018). It is also been shown that the mTOR pathway is involved in the antidepressant effect of creatine (Cunha et al. 2016). This is another piece of evidence that the screened dysregulated genes enriched in the mTOR pathway have great potential in the development of PTSD, which deserves in‐depth study. Our analysis also highlighted the role of hub genes in pathways such as nicotine addiction and dopaminergic synapse. Nicotine use has been associated with an increased risk of developing PTSD, and exposure to nicotine can exacerbate distressing memories related to traumatic events (Breslau, Davis, and Schultz 2019; Kutlu et al. 2018). A study of a nationally representative sample of US adults also found that nicotine dependence may increase the risk of developing PTSD after exposure to trauma (Ibrahim, Le Foll, and Hassan 2022). Moreover, the role of dopamine in the physiopathology of PTSD has been broadly investigated. Dopaminergic dysfunction and a decrease in dopamine metabolite levels in the cerebrospinal fluid have been observed in patients with PTSD (Rytwinski et al. 2013; Wolf et al. 2014; Shoji and Miyakawa 2021). What is more, PTSD patients have been found to have altered expression levels of dopamine‐related genes and proteins, leading to regional abnormalities in dopamine transmission and metabolism. Dopamine plays a crucial role in fear memory processes, and dopaminergic treatments have been shown to alleviate PTSD‐like symptoms in rodent models (Lin et al. 2020; Malikowska‐Racia et al. 2019). In addition, PTSD patients have been found to have altered expression levels of dopamine‐related genes and proteins, leading to regional abnormalities in dopamine transmission and metabolism (Zhou et al. 2021). Given the challenges of studying PTSD in human subjects, appropriate animal models are essential for validating the roles of the key genes identified in this study.

Based on the animal model data from our dataset, we constructed an SS mouse model to assess the role of target genes in PTSD‐like symptoms (Muhie et al. 2015). Behavioral tests, including OFT and EMPT, demonstrated significant behavioral changes in the SS group, validating the success of the SS animal model. The SS group showed reduced exploration of the new environment, increased levels of anxiety, and significant depression‐like behavior, consistent with the notion that stress exposure can induce anxiety‐like behavior and contribute to PTSD. Furthermore, our integrated analysis, combining data mining, bioinformatics, and qRT‐PCR, identified *Gabra1*, *Gabra2*, *Grin2b*, and *Drd2* as potential biomarkers for diagnosing and treating PTSD. These hub genes are primarily involved in the neuroactive ligand‐receptor interaction pathway, an area that has not been extensively studied and warrants further exploration. The *GABRA1* and *GABRA2* genes encode subunits for the GABA‐A receptor. *GABRA1*, located on human chromosome 5q34‐q35, has been implicated in psychological disorders (Johnson et al. 1992; Serretti et al. 1999). Anxiety has been linked to GABAAR (Olivier, Vinkers, and Olivier 2013). Following exposure to juvenile and subsequent adult stress, distinct changes in the expression of *Gabra1* and *Gabra2* subunits in the basolateral amygdala (BLA) of rats were described (Shlomit et al. 2008). Moreover, increased expression of *Gabra2* in the AMY has also been reported in models of early‐life stress (Gondré‐Lewis et al. 2016). Similarly, related studies suggest that Gabra may also be involved in the pathophysiology of major depressive disorder (MDD) and that depression‐like symptoms are an extremely important part of the symptoms associated with PTSD (Henkel et al. 2004). The *Grin2b* gene encodes subunits of the *N*‐methyl‐d‐aspartate (NMDA) receptor channel, which is involved in synaptic plasticity and drug dependence (Kelley 2004; Hyman, Malenka, and Nestler 2006). The pathway related to *Grin2b* is nicotine addiction. *Grin2b* polymorphisms have been associated with addiction. A previous study reported an association between *Grin2b* polymorphism and the development of nicotine dependence (Grucza et al. 2010). *Grin2b* is regarded as a promising genetic marker for MDD susceptibility, which serves as a genetic predictor for treatment‐resistant depression (C. Zhang et al. 2014). Although in our study Grin2b expression was significantly increased in HIP and AMY in SS group rats, more relevant experiments and studies are also lacking to further validate our results; therefore, the relationship between Grin2b, nicotine dependence, and PTSD needs to be further investigated. However, the relationship between *Grin2b*, nicotine addiction, and PTSD remains underexplored and requires further investigation. Dopamine neurotransmission is crucial for spatial learning and synaptic plasticity, and disruptions in this system are linked to psychiatric disorders (Kempadoo et al. 2016). Dopamine receptors are divided into two main subtypes: Drd1‐like and Drd2‐like, which work together to regulate neurotransmission (Sun, Luquet, and Small 2017). *Drd2*, part of the Drd2‐like group, is associated with reward circuits (Noble 2000). In our study, Drd2 expression was increased in the AMY of rats in the SS group, similar to that in the rodent studies have demonstrated the involvement of D2 receptors in the BLA in the learning of fear extinction, with local D2 receptor antagonists affecting extinction learning in rat models of PTSD (Shi et al. 2017). *Drd2* polymorphisms have also been linked to PTSD risk (Hoxha et al. 2019). In summary, bioinformatic analyses offer insights into the mechanisms underlying PTSD development and identify potential targets for future treatments.

Certain limitations to this study warrant further investigation. First, we analyzed only specific regions of the brain (HIP and AMY) and a subset of the dataset, which may have limited our findings. Future research will involve examining additional brain regions and datasets. Second, further in vitro experiments are necessary to validate our results. In future studies, we need to include more behavioral tests to support our findings. Third, there was a lack of verification of protein expression corresponding to the identified genes, which will be addressed in future studies. Finally, our current data sources and animal models are derived from mice, and we will continue to incorporate rat and human data into the study for future experiments. In the meantime, expanding our exploration to include more brain regions and mechanisms will enhance our understanding of PTSD development.

## 5 Conclusions

Our bioinformatic analysis of brain transcriptome profiling data from a mouse model simulating PTSD characteristics identified four key genes potentially involved in the development of PTSD. Meanwhile, exposure to SS successfully induced PTSD‐like symptoms in mice, including a range of adverse emotional responses such as depression‐like, anxiety‐like, and panic‐like behaviors (endophenotype). These results provide molecular evidence suggesting that the identified genes play a significant role in PTSD development. In addition, our findings highlight physiological deficits in reward circuits associated with psychiatric disorders, including bipolar disorder, MDD, attention‐deficit/hyperactivity disorder, and PTSD. This study underscores the potential of bioinformatics in elucidating the mechanisms underlying PTSD and points to promising avenues for developing more effective treatments in the future.

## Author Contributions

**Lifen Liu**: writing–original draft, methodology, data curation, investigation. **Yang Liu**: data curation, methodology. **Rui Li**: methodology, data curation. **Yue Teng**: data curation, methodology. **Shuyang Zhao**: data curation. **Jinhong Chen**: conceptualization, project administration. **Changjiang Li**: conceptualization, supervision. **Xinyu Hu**: methodology, data curation, formal analysis, visualization, validation.

## Ethics Statement

This study was performed in line with the principles of the Declaration of Helsinki and approved by the Shandong Second Medical University Animal Care and Use Committees.

## Consent

Informed consent was obtained from all individual participants included in the study.

## Conflicts of Interest

The authors declare no conflicts of interest.

### Peer Review

The peer review history for this article is available at https://publons.com/publon/10.1002/brb3.70243.

## Data Availability

Data will be made available on request.
